# Regenerative Effects of Exosomes-Derived MSCs: An Overview on Spinal Cord Injury Experimental Studies

**DOI:** 10.3390/biomedicines11010201

**Published:** 2023-01-13

**Authors:** Giovanni Schepici, Serena Silvestro, Emanuela Mazzon

**Affiliations:** IRCCS Centro Neurolesi “Bonino-Pulejo”, Via Provinciale Palermo, Contrada Casazza, 98124 Messina, Italy

**Keywords:** exosomes, spinal cord injury, mesenchymal stromal/stem, blood-spinal cord barrier integrity

## Abstract

Spinal cord injury (SCI) is a devastating condition usually induced by the initial mechanical insult that can lead to permanent motor and sensory deficits. At present, researchers are investigating potential therapeutic strategies to ameliorate the neuro-inflammatory cascade that occurs post-injury. Although the use of mesenchymal stromal/stem (MSCs) as a potential therapy in application to regenerative medicine promoted anti-inflammatory and neuroprotective effects, several disadvantages limit their use. Therefore, recent studies have reported the effects of exosomes-derived MSCs (MSC-EXOs) as an innovative therapeutic option for SCI patients. It is noteworthy that MSC-EXOs can maintain the integrity of the blood-spinal cord barrier (BSCB), promoting angiogenic, proliferative, and anti-oxidant effects, as well as immunomodulatory, anti-inflammatory, and antiapoptotic properties. Therefore, in this study, we summarized the preclinical studies reported in the literature that have shown the effects of MSC-EXOs as a new molecular target to counteract the devastating effects of SCI.

## 1. Introduction

Spinal cord injury (SCI) is a disabling condition that causes partial or complete sensorial and motor deficits. Usually, SCI is the result of an initial mechanical insult, followed by a cascade of structural and neuroinflammatory changes. To ameliorate the damaging events that occur after SCI, researchers have been evaluating the potential of mesenchymal stromal/stem cells (MSCs) as therapeutic agents in application to regenerative medicine. Despite the fact that experimental studies that have used MSCs have proven their beneficial effects on SCI, there are several limitations regarding the application of MSCs as therapeutic agents for humans. Therefore, it is necessary to identify new agents for regenerative medicine application [1].

MSCs exert their therapeutic effects through the release of extracellular vesicles (EVs), a group of membranous vesicles that are cell-derived and 30–1000 nanometer in size, composed of lipid bilayers and secreted into the extracellular space [2]. EVs have been found in biological fluids, including cerebro-spinal fluid (CSF), blood, urine breast milk, saliva, amniotic fluid, and synovial fluid [3]. In addition to MSCs, EVs are also produced by several cell types, such as B cells, NK cells, T cells, erythrocytes, platelets, epithelial cells, endothelial cells, dendritic cells, neurons, oligodendrocytes, Schwann cells, muscle cells, cancer cells and embryonic cells [4]. Based on their size and biogenesis, three comprehensive classes of EVs are known: exosomes, microvesicles (MVs) and apoptotic bodies. Apoptotic bodies are vesicles of 50–2000 nm in size that result from cell death. MVs are vesicles of 150–1000 nm in diameter, obtained by direct budding from cytoplasmic membrane. Exosomes are endosomal vesicles of size 40–150 nm in diameter generated by the fusion of multivesicular bodies (MVBs) with the cell membrane that are released into the extracellular environment [4,5].

It was demonstrate that EVs exert the capacity of transferring biological molecules such as proteins, nucleic acids and lipids without direct cell-to-cell contact [6]. Thanks to their inclusion, morphology and ability to act as carriers to reach injury sites through biological barriers, EVs could be useful for the diagnosis and treatment of diseases [7]. Consequently, advancing technologies in regenerative medicine have led researchers to the isolation and application of exosomes-derived MSCs (MSC-EXOs). 

In this regard, it has been demonstrated that MSC-EXOs exerts MSCs physiological functions including anti-inflammatory, restorative, regenerative and immunomodulatory potential. Therefore, due to their specific properties, researchers have investigated MSC-EXOs as a potential therapeutic application for the treatment of permanent disability conditions such as SCI [7,8].

The aim of this review is to summarize the preclinical studies that have reported the effects of MSC-EXOs as a possible therapeutic strategy for SCI.

## 2. Methodology

Publications between 2017 and 2022 are taken into consideration for this review. PubMed and Scopus were used to retrieve the publications that corresponds to the keywords: “MSCs derived exosomes” and “spinal cord injury”.

**Figure 1:**
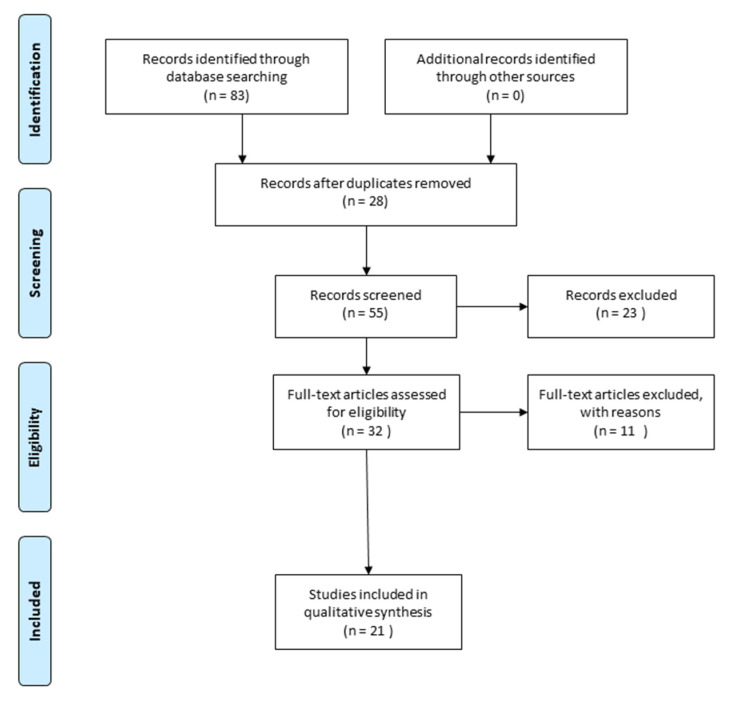
The Prisma flow diagram illustrates the methodology that was used to select the in vivo and in vitro studies used for the writing of the review. Duplicate articles were excluded from the total of the studies that were found. Conversely, articles that highlight the role of MSCs derived exosomes in promoting injury repair and restoring functional deficits are described (The PRISMA Statement is published in [9].

Studies that have evaluated the role of MSCs-derived exosomes in promoting SCI repair and reducing functional deficits were selected. The Prisma flow diagram illustrates the article selection process (Figure 1).

## 3. Spinal Cord Injury (SCI)

SCI is a devastating injury to the spinal cord that leads to temporary or permanent changes into the spinal cord, as well as the partial or complete loss of motor, autonomic and sensory function [10,11]. SCI is common in males below 30 years of age [12,13]. The hallmarks of SCI are generally paralysis (paraplegia or quadriplegia), with sensory dysfunction below the injury level [14]. It has been demonstrated that SCI can induce the loss of connection between the brain and peripheral nervous system, in turn negatively influencing the majority of basic bodily functions, including breathing, sexual function, hormone release, as well as bowel and bladder functions [15].

Mechanical insult due to physical forces, including compression or contusion, can lead to the transection or stretching of the spinal column with consequent spinal cord disruption, thus promoting the primary injury [16,17,18]. 

“Primary injury” is an irreversible process that occurs immediately following injury to the spinal cord, leading the axonal membranes to rupture and the release of materials that inhibit axonal regeneration, including the neurite outgrowth inhibitor protein A, oligodendrocyte myelin glycoprotein, chondroitin sulfate proteoglycan and myelin-associated glycoprotein [19,20]. 

**Figure 2:**
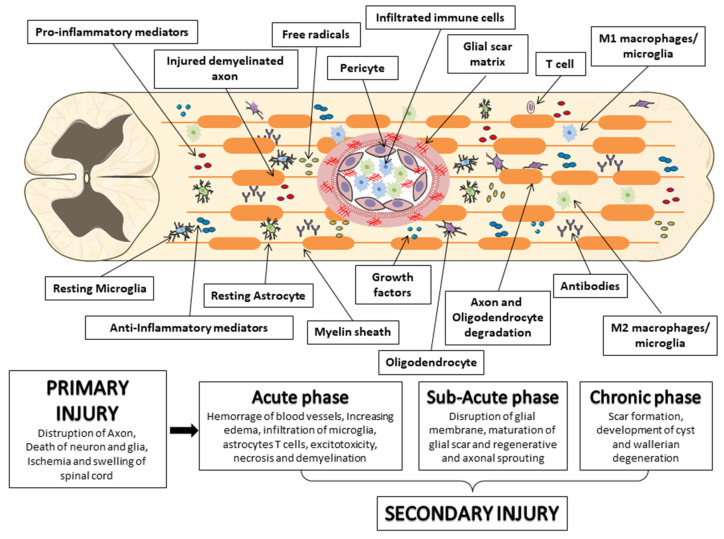
Pathophysiology of spinal cord injury (SCI). This schematic diagram illustrates the phase of SCI and its pathophysiology. Immediately after primary injury, the activation of resident astrocytes and microglia, and the subsequent infiltration of blood immune cells, induce an important neuroinflammatory response. This acute neuroinflammatory response plays a key role in the secondary injury mechanisms in the sub-acute and chronic phases that lead to cell death and tissue degeneration, as well as the formation of the glial scar, axonal degeneration and demyelination. During the acute phase, monocyte-derived macrophages occur in the central area of the injury to scavenge tissue damage. In these phases, the loss of oligodendrocytes leads to axonal demyelination, followed by spontaneous remyelination. Instead, astrocytes and pericytes, normally present in the spinal cord parenchyma, after damage proliferate and migrate to the site of injury and contribute to the formation of the glial scar. The image was created using the image bank of Servier Medical Art (Available online: http://smart.servier.com/, accessed on 1 December 2022), licensed under a Creative Commons Attribution 3.0 Unported License (Available online: https://creativecommons.org/licenses/by/3.0/, accessed on 1 December 2022).

The subsequent phase of SCI is known as “secondary injury”, in which a cascade of events are triggered, leading to progressive neuronal tissue damage and the exacerbation of neurological deficits [21]. The secondary lesion is divided into an acute, a subacute, and a chronic phase [22]. The acute phase is characterized by progressive hemorrhage, edema, ischemia, thrombosis, an increase in oxidative stress, apoptosis, cell necrosis, as well as the release of inflammatory cytokines due to the rupture of the blood-spinal cord barrier (BSCB) [11]. Secondary injury negatively regulates cell survival, leading to an increase in the lesion into the spinal cord in the rostrocaudal directions [23]. Indeed, it has been shown that, among the mechanisms of secondary injury, the major contribution is provided by inflammation due to macrophages, T-cells, microglia and neutrophils infiltration into the site of the lesion, thus promoting the blood-marrow barrier rupture [24]. These cells generate an inflammatory environment mediated by the release of interleukin (IL)-1β, IL-6, and tumor necrosis factor-α (TNFα). It is noteworthy that the inflammatory cytokines reach their peak 6–12 h after injury and stay in circulation for four days [25]. The acute phase is followed by the subacute phase; this is characterized by ionic homeostasis imbalance, which is responsible for an increase in intracellular calcium, which in turn promotes mitochondrial dysfunction and cell death [26,27]. In particular, oligodendrocyte death promotes the axon demineralization process [28]. The mitochondrial changes induce DNA oxidative damage, oxidation of proteins, and lipid peroxidation, leading to reactive oxygen species (ROS) and reactive nitrogen species (RNS) production with further exacerbation of the tissue damage [29]. The expansion of the damage prompts the chronic phase wherein the activation of astrocytes produces excessive levels of the extracellular matrix and increases neuronal apoptosis. This process triggers cystic cavity formation, axonal death and the maturation of the glial scar [30,31]. Consequently, scar formation, tissue necrosis, and cavity can interfere with cell regenerative processes, such as axonal regeneration, which compromises the functional recovery and therapeutic regenerative potential [32]. While in the early phase of secondary injury, the glial scar exerts a positive role in containing inflammation, both removing debris and regenerating BSCB. In the later phase of injury, axonal growth inhibitors and scars can interfere with neuronal regeneration (Figure 2) [33].

Although advances in SCI management have led to an improvement in patients’ quality of life, their recovery remains limited [34]. 

In this regard, the current treatments, including surgical decompression [35], spinal cord pressure monitoring [36], corticosteroids [37,38] and hemodynamic therapy [39], do not completely restore the function of the damaged spinal cord; thus, it is necessary to find new therapeutic treatments for SCI management, such as MSC-EXOs. 

## 4. MSC-EXOs

Exosomes are natural nanomaterials enclosed by a lipid bilayer membrane that contains phosphatidylserine, cholesterol, sphingomyelin and ceramide. It is noteworthy that exosomes play an important role in cellular communication through the transport of genetic information and bioactive substances, including proteins, nucleic acids and lipids, to create paths for communication between cells. In this regard, it was reported that exosomes could maintain the homeostasis of the CNS crossed with the BBB and communicate through the miRNA proteins in the neurovascular unit [40]. 

Exosomes are widely distributed in some body fluids, such as blood, cerebrospinal fluid, urine, amniotic fluid, pericardial effusion, milk and saliva [41]. Additionally, exosomes express surface proteins, including membrane transport/fusion proteins and heat shock proteins (HSPs). Despite electron microscopy being the golden standard method for exosome identification, the presence of CD9, CD63, and CD81 favors their identification [42]. Several techniques for the isolation of exosomes from different sources have been developed [43]. Ultracentrifugation is the most used isolation method and is based on the density difference and particle size. Additionally, it is a simple and cost-effective method that allows for isolating a high amount of exosomes [43,44]. The size-exclusion chromatography and ultrafiltration are isolation methods that lead to the biomolecule’s separation, according to their size [45]. Additionally, immunocapture techniques are exosome isolation methods based on the interactions between the antibodies and proteins present in the exosome’s surface [46]. Polymer precipitation is an isolation method based on the solubility exosome changes [47]. Another method used to isolate and purify exosomes involves the use of microfluidic technologies in order to obtain high purity and sensitivity [43].

As exosomes are membrane-coated nanoparticles, their use as vehicles for drug delivery, as well as in immune modulation and tissue regeneration, are being investigated. Hence, techniques that employ the modification of exosomes to increase their targeting efficiency and cross-biological membranes could be useful for biomedical applications. At present, the gold standard technique for modifying the surface of nanoparticles is poly (ethylene glycol) (PEG) coating, which has led to improved pharmacokinetics through the stability and immunogenicity of the nanoparticles in vivo compared to uncoated nanoparticles. Despite this, it was reported that PEG-coated nanoparticles mediate complement activation and recognition by PEG-specific IgM antibodies with an increase in blood clearance by the liver and hypersensitivity in vivo. Therefore, integrated cell cloaking nanotherapeutics that exploit the biomimetic intrinsic properties of cell membranes could enhance the increment of the nanoparticles in the target tissue. In this regard, it was reported that macrophage membrane-coated nanoparticles improved their accumulation in inflammatory sites. Additionally, prolonged circulation in vivo was also shown in red blood cell membrane-coated nanoparticles. Moreover, to improve the integrated cell cloaking nanotherapeutic techniques for potential clinical applications, the combination of genetic, chemical and physical engineering and nanotherapeutic platforms, including mesoporous silica nanoparticles, magnetic nanoparticles and metal-organic framework, is providing promising results both in regenerative medicine and cancer immunotherapy [48].

Exosome biogenesis is achieved through plasma membrane invagination and the generation of intracellular MVBs with intraluminal vesicles (ILVs). The endocytic pathway, which involves the donor cell, is followed by the transport of intravesicular and transmembrane proteins from the Golgi complex, to lead to the formation of early endosomes [49]. Subsequently, the differentiation and maturation of early endosomes into late endosomes occurs [50]. Morphological and physical changes can identify the transition into late endosomes. In this regard, it has been reported that early endosomes show a tubular shape and are found in the external portion of the cytoplasm. In contrast, late endosomes are spherical and located near the nucleus [51]. Additionally, late endosomes are degraded by binding to lysosomes, plasma membranes or autophagosomes to release the ILVs into the extracellular environment as exosomes [52]. The interaction of exosomes with the recipient cells can occur through their surface receptor molecules and ligands. It has been demonstrated that, after secretion, some exosomes stay on the cell membranes of the donor cells, while the remaining ones interact with the recipient cells [53]. Exosome internalization is a process related to caveolae, raft or clathrin-dependent endocytosis. Additionally, it has been reported that phagocytosis and micropinocytosis can also be considered as methods of internalization. Therefore, exosome internalization that targets recipient cells can be interesting as a potential therapeutic application in regenerative medicine [54,55]. In comparison to MSCs, exosomes have both higher safety and stronger plasticity, deeming them potentially useful in clinical application to Central Nervous System (CNS) injuries [56,57].

As it has been demonstrated that the differentiation of MSCs towards a neural lineage in the damaged brain only includes a limited number of cells, the regenerative potential of MSCs could be more related to their paracrine activity, which is performed by exosomes [44,45].

In addition to the features of MSCs, MSC-EXOs are known for their immunomodulatory, anti-inflammatory, antiapoptotic, angiogenic, proliferative and antioxidant effects [58]. Moreover, MSC-EXOs offer several benefits, including accessible storage, high stability and low immunogenicity [59,60]. MSC-EXOs are cystic vesicle cup-shaped structures with a diameter of 30–100 nm, containing lipids, proteins and RNA [13,14]. The lipid bilayer is important in stabilizing biological activities and maintaining the integrity of the exosomes, while protein modification on the surface leads to an improvement in targeting and recognition of the exosomes. Additionally, the presence of nucleic acids, such as microRNAs (miRNAs), is important in intercellular communication [61,62].

Despite their biogenesis being common to other sources, it has been reported that MSC-EXOs show no differences in terms of their isolation, conservation and morphological characteristics. Moreover, MSC-EXOs have demonstrated the ability to produce more exosomes than other cell lines [15,16]. In addition to the common surface markers CD9 and CD81, MSC-EXOs express some specific adhesion molecules, including CD29, CD44 and CD73, present on the MSCs membrane [17].

Therefore, based on their paracrine effects, MSC-EXOs could be useful to improve functional recovery, as well as being used as a cell-free therapeutic strategy [63].

## 5. MSC-EXOs as a Potential Therapeutic Tool in SCI

MSC-EXOs are easier to collect and store and possess poor ethical restrictions compared to MSCs. For this reason, researchers have been evaluating the potential therapeutic effects of MSC-EXOs in SCI experimental studies [64]. Several experimental studies have reported that MSC-EXOs can exert anti-inflammatory and anti-apoptotic effects, promote axonal regeneration and macrophage polarization, as well as protect the BSCB from spinal cord damage [65,66,67]. The functional recovery of SCI patients is related to pro-inflammatory and anti-inflammatory environments, as well as relative levels of pro-inflammatory cytokines and anti-inflammatory factors [68,69]. In this regard, it has been demonstrated that the intravenous injection of human umbilical cord MSC-EXOs inhibited the expression of proinflammatory cytokines IL-1β, IL-6 and the formation of scars, and promoted motor function recovery in a SCI rat model [70].

Neuroinflammation plays an important role in secondary injuries of SCI. It has been demonstrated that neuroinflammation leads to the activation of resident immune cells and is mediated by a protein complex-inflammasome known as the nucleotide-binding domain-like receptor protein 3 (NLRP3) inflammasome. NLRP3 is located in the cytoplasm and regulates the natural immune response [71]. It is noteworthy that an increase in NLRP3 inflammasome activity in a SCI mice model has been reported [72]. Additionally, the inhibition of NLRP3 inflammasome activation led to functional recovery in SCI rats, thus confirming the important role of NLRP3 inflammasome in the control of the inflammatory events that occur in SCI [72,73]. Similarly, it has been shown that the systematic administration of epidural fat MSC-EXOs induced injury reduction and improved neural function recovery in SCI rats, according to a molecular mechanism that led to the suppression of NLRP3 inflammasomes activation and a reduction in inflammatory cytokines. Additionally, an increase in B cell lymphoma-2 (Bcl-2) and a decrease in Bcl-2 associated X protein (Bax) was observed [65].

As SCI leads to the activation of both the classic and alternative complement pathways, thus exacerbating the inflammatory reaction, the inhibition of nuclear factor kappa-light-chain-enhancer of activated B cells (NF-κB) may be a possible strategy for minimizing inflammation in secondary injury and for promoting functional recovery [74]. Consequently, the interaction of complements C1q and C3 to the NF-κB signalling pathway could explain this [75]. Additionally, it has been observed that the C3 complement protein a (key marker of A1 astrocytes) upregulates NF-κB in a dependent manner [76]. In this respect, in a SCI rats model, the administration of bone marrow MSC-EXOs led to a reduction in A1 astrocytes through the downregulation of NFκB P65 [77].

In addition, macrophages are involved in the inflammatory process that occurs in secondary injuries of SCI. Researchers have demonstrated the potential therapeutic effect of MSC-EXOs in macrophage polarization [78]. It is known that macrophages can switch from the pro-inflammatory phenotype M1 to the anti-inflammatory M2 one [79]. Following injury, myelin damage at the initial SCI site leads to the infiltration of the macrophage via chemotaxis [80]. M1 macrophages induce the production of pro-inflammatory cytokines, including TNF-α, IFN-γ, IL-6, ROS and nitric oxide, with harmful effects to the injured spinal cord [78]. In contrast, M2 macrophages exert anti-inflammatory effects through IL-10 release and promote axonal regeneration [80,81]. Hence, the persistence of M1 macrophages induces a severe inflammatory state in SCI and causes further damage to the injured spinal cord. Consequently, it is necessary promote strategies that lead to the polarization of macrophages from the M1 phenotype to the M2 type [82]. The intravenous administration of MSC-EXOs targets M2 macrophages in the injured spinal cord in order to release EVs by MSCs and to improve SCI [83].

It is worth noting that the BSCB plays an important role both in the normal function of the nervous system and in maintaining its integrity. The BSCB is formed by the basement membrane, capillary endothelial cells, astrocyte foot processes and pericytes. Therefore, the BSCB could be a potential therapeutic target for SCI [84]. After SCI, the rupture of the BSCB induces the destruction of the blood vessels at the site of injury and causes a consequent increment both of permeability and toxic materials into the injured spinal cord, leading to edema and neuronal cell death [85]. Additionally, pericytes, as a part of the neurovascular unit, are involved in the formation and maintenance of the blood vessels’ properties, as well as BSCB integrity [86]. It is noteworthy that pericytes and several vascular wall cells could induce vessel wall fragility and, thus, the development of spinal vascular shunts and arteriovenous malformations (AVMs), as well as intracerebral hemorrhage [87]. Pericytes play an important role in maintaining vessel stability and angiogenesis through crosstalk with angiopoietin signalling. In this regard, it was reported that Angiopoietin-2, in the presence of the vascular endothelial growth factor (VEGF), can promote vessels formation [88]. The therapeutic potential of MSCs-EXOs in SCI could also occur via the NF-κB p65 pathway, thus inhibiting the migration of pericytes and, promoting BSCB integrity, axonal regeneration and improved motor function, as well as leading to a reduction in neuronal cell apoptosis [66]. Additionally, it has been reported that pericyte from EXOs reduced cell apoptosis, improved microcirculation and protected the BSCB in SCI mice [89].

The importance of miRNAs, such as miRNA-21, miRNA-133 and miRNA-126, as potential therapeutic targets for SCI has recently been proposed [90]. 

Therefore, thanks to their characteristics, EXOs could cross the BSCB, enhancing the effects of miRNAs, and potentially be used as carriers to miRNAs at the SCI site [91].

Moreover, during tissue damage, an increase in miRNA-21 expression has been highlighted, with a reduction in neuronal apoptosis via the phosphatase and tensin homolog (PTEN)-protein kinase B (Akt) signalling pathway, as well as the modulation of the expression levels of protein-related apoptosis, including Bcl-2, Bax, caspase-3 and caspase-9 [92,93]. Additionally, it has been reported that the downregulation of miRNA-133b led to a reduction in neuronal axonal regeneration and prevented the recovery of motor function [94]. It is worth noting that a decrease in miRNA-126 after SCI has been shown, while an increase in miRNA-126 led to a reduction in inflammation and improved functional recovery and angiogenesis [95]. 

## 6. MSC-EXOs as a Potential Therapeutic Tool in SCI Experimental Studies

Several experimental studies have investigated the effects of MSC-EXO as a potential therapeutic strategy for SCI.

Sung et al. evaluated the efficacy of MSCs-EXOs from human epidural adipose tissue (hEpi AD), isolated by tangential flow filtration and characterized by flow cytometry, in addition to nanoparticle tracking analysis. HEpi ADMSCs-EXOs were administrated intravenously, both at low and high concentrations, in SCI-Sprague Dawley (SD) rats and induced by the compression method. The study results demonstrated that the hEpi ADMSCs-EXOs led to a reduction in the inflammatory responses, as well as improved spinal functions. Histopathological and immunohistochemical analyzes of Iba-1 and glial fibrillary acid protein (GFAP) in rat spinal cords demonstrated that the administration of high and low concentrations of hEpi ADMSCs-EXOs reduced neuroinflammation. Overall, an increase in the brain-derived neurotrophic factor (BDNF) level in the spinal cord of rats after the administration of high MSCs-EXOs concentrations was significantly reduced in the SCI vehicle group. In addition, VEGF, an important neurotrophic factor for the survival of spinal neurons, was higher in the spinal cords of the treated mice. Additionally, a reduction in the pro-inflammatory cytokines IL-1β, IL-2 and TNF-α in the MSCs-EXOs groups was highlighted. It is noteworthy that mRNA sequencing analysis of the spinal cord tissue demonstrated that the administration of hEpi ADMSCs-EXOs reduced the SCI-induced inflammatory responses by targeting the immune response and neurogenesis-related genes, thus suggesting that MSCs-EXOs could be useful to improve neurological recovery in SCI [96].

In contrast, Zhou et al. evaluated the effects of bone marrow (BM) MSC-EXOs both in vivo and in vitro. In order to perform the in vivo model, the authors used SD rats induced with the impact method and subsequently treated with BMMSC-EXOs. The in vivo results demonstrated that the transplant of the BMMSC-EXOs led to a reduction in myelin loss and neuronal cell death, and improved both the myelin arrangement and locomotor functional recovery. Moreover, the transplant of the BMMSC-EXOs reduced edema and caspase-1 expression, and inhibited the pericyte pyroptosis and IL-1β release, thus maintaining BSCB integrity. To confirm the in vitro results, microvascular pericytes isolated from a rat’s spinal cord were induced with IFN-γ 100 ng/mL plus TNF-α; 10 ng/mL and transfected with lipopolysaccharide (LPS) 1 μg/mL, premixed with Lipofectamine, as well as co-incubated with or without adenosine triphosphate (ATP) 5 mM. Additionally, to test the protective effect of the MSCs-EXOs, pericytes were co-incubated with or without BMMSCs-EXOs (100 μg/mL). The exposure of pericytes to IFN-γ + TNF-α + LPS + Lipofectamine + ATP led to the upregulation of pro-caspase 1 and Nod1 inflammasome, as well as the release of IL-1β. In addition, the treatment with BMMSCs-EXOs effectively inhibited Nod1 inflammasome and pyroptosis in the pericytes, promoting the inhibition of IL-1β release and the upregulation of pro-caspase 1. Therefore, overall, the data suggest that the use of BMMSCs-EXOs improved the integrity of the BSCB after SCI through pericyte pyroptosis inhibition [97].

Zhang et al. also demonstrated the potential therapeutic effects of exosomes derived from human placental MSCs (hPMSCs-EXOs) in SCI. The hPMSCs-EXOs, extracted by sequential centrifugation, displayed proangiogenic effects both in vitro and in vivo. Male mice induced by the contusive SCI model were administered with hPMSCs-EXOs (200 μg/μL) into the SCI epicenter. The in vivo results reported that the transplant of hPMSCs-EXOs led to improved locomotor and sensory function recovery, as well as promoting the new vessel formation and angiogenesis, as shown by a subset of endothelial cells near the injury site that took up the labelled hPMSCs-EXOs. Additionally, to develop the in vitro model and simulate ischemia in SCI, Human Umbilical Vein Endothelial Cells (HUVECs) were cultivated in oxygen-glucose deprivation (OGD) conditions scratching was performed, and they were divided into experimental groups. The in vitro results highlighted that treatment with hPMSCs-EXOs promoted tube formation by endothelial cells, thus confirming the proangiogenic effects of hPMSCs-EXOs. Although is not yet known whether endothelial cells can uptake MSCs-EXOs in the injured area, it is possible that endothelial cells can only uptake MSCs-EXOs at certain stages of their development. Therefore, further studies are required to elucidate the underlying mechanisms [98].

PTEN-AKT-mTOR pathway activation could also promote neurogenesis and axonal regeneration, leading to a reduction in glial scarring and thus be potentially useful for SCI treatment. As miR-26a could activate the PTEN-AKT-mTOR pathway, Chen et al. investigated the effects of BM-MSCs transfected with the mimics of miR-26a, from which the exosomes were harvested. After transfection, the expression level of miR-26a in the exosomes was significantly increased. Then, PC12 cells were incubated with miR-26a overexpressing exosomes (BMMSCs-EXOs-miR-26a) for 48 h. In vitro, BMMSCs-EXOs-miR-26a promoted neurofilament generation in the PC12 cells by downregulating PTEN expression and consequently increasing the phosphorylation of Phosphoinositide 3-kinases (PI3Ks), AKT and the mammalian target of rapamycin (mTOR) proteins. Targeting the PTEN-AKT-mTOR pathway increased NF generation and nerve regeneration. In addition, in vivo, in the SCI rat model, the injection of BMMSCs-EXOs-miR-26a reduced the injury in the damaged area and ameliorated the functional recovery. Following SCI, miR-26a-overexpressing BMMSC-EXOs improved axonal regeneration, as also shown by the upregulation of neurofilament and β-tubulin-3. Therefore, miR-26a could activate the PTEN-AKT-mTOR pathway and promote axonal regeneration and neurogenesis [99].

Recently, several studies have investigated the potential role of MSCs-EXOs as a miRNAs source in SCI experimental studies. In this regard, Li et al. studied the effects of BMMSCs-derived EXOs transfected with miR-544. Following extradural compression to induce the SCI model, SD rats were treated with BMMSCs-EXOs containing miR-544. The BMMSCs-EXOs transfected with miR-544 improved the neural functional recovery in SCI rats. Additionally, it has been evidenced that overexpressing miR-544 can alleviate the inflammatory response after SCI, evaluated by proinflammatory cytokines reduction including TNF-α, IL-1α, IL-17B and IL-36β [100].

As miRNAs represent the most abundant nucleotides in exosomes, they are receiving increasing attention from researchers. In this regard, Wang et al. identified the expression profiles of exosomal miRNAs derived from human umbilical cord mesenchymal/stromal stem cells (hUCMSCs). MiR-199a-3p and miR-145-5p were the most abundant miRNAs identified in the hUCMSC-EXOs. Furthermore, bioinformatic analysis showed that these miRNAs regulate TrkA turnover, as there seems to be a direct relationship between miR-199a-3p and Cblb, miR-145-5p and Cbl. In this regard, the role of exosomal miR-199a-3p/145-5p on neuronal differentiation through the regulation of the *Cblb* and Cbl genes was explored. To evaluate the synergistic effects of miR-199a-3p/145-5p in EXOs, hUC-MSCs were co-transfected with miR-199a-3p/145-5p antisense RNA and the EXO were isolated. To simulate the inflammatory environment and promote the neurite outgrowth that occurs after SCI, an injury model was developed. The PC12 cells were induced with LPS 11 μg/mL, highlighting both the reduction in cell differentiation and miR-199a-3p/145-5p expression. It is noteworthy that when the PC12 cells were pre-treated with hUCMSCs-EXOs co-transfected with miR-199a-3p/145-5p, an increase in PC12 cell differentiation by modulation of the nerve growth factor (NGF)/TrkA pathway differentiation was observed. Moreover, the overexpression of neuronal markers, including β-tubulin-3, heavy-chain neurofilament (NF-H) and Neuronal Nuclei (NeuN) was shown. On the contrary, the knockdown of miR-199a-3p/145-5p in MSCs-EXOs partially abolished the protective effect of the exosomal miRNAs in the PC12 cells. In addition, bioinformatic analysis followed by Western blotting and QRT-PCR analysis demonstrated that miR-199a-3p and miR-145-5p targeted, respectively, the Cblb and Cbl genes, leading to the modulation of TrkA ubiquitination. To confirm the data obtained, the authors performed the study in an in vivo model of SCI. In addition, the in vivo results demonstrated that the injection of EXOs led to an antiapoptotic effect at the lesion site; this effect was partially attributed to the exosomal miR-199a-3p/145-5p that upregulated TrkA expression. Additionally, it has been reported that hUCMSCs-EXOs co-transfected with miR-199a-3p/145-5p infusion improved locomotor function in SCI rats and reduced inflammation [101].

Obesity is considered a metabolic disorder that reshapes the MSCs and their EVs; in this regard, Ji et al. evaluated the protective effects of miR-21 transfected in MSCs-EXOs derived from obese rats. To perform the study, SD rats were induced by laminectomy and were administrated with MSCs-EXOs 24 h after surgery. It has been demonstrated that MSCs-EXOs derived from obese rats did not exert a beneficial effect on the recovery of hindlimb locomotor function and a decrease in miR-21 levels was also observed compared to the group induced with MSCs-EXOs from the control rats. The involvement of miR-21 in SCI’s protective effects has been demonstrated by treating MSCs derived from obese rats with miR-21 mimics. MSC-EXOs isolated from obese rats transfected with miR-21 mimic decreased cell apoptosis and injury, and improved locomotor function. Additionally, it has been reported that obesity-related insulin resistance promoted miR-21 deficiency in MSCs-EXOs purified from obese rats. Therefore, it has been suggested that miR-21 could be a potential target for SCI treatment [102]. In addition, Kang et al. investigated the effect of miR-21 on SCI, as well as their underlying molecular mechanisms. It has been demonstrated that miR-21 targets the phosphatase and tensin homolog (PTEN). Indeed, after injury, an increase in miR-21 expression and a consequent reduction in PTEN was observed. To induce an experimental study and investigate the role of miR-21, the authors used MSC-EXOs transfected by miR-21 or PTEN siRNA. The MSC-EXOs transfected by miR-21 or PTEN siRNA led to a reduction in neuronal loss and improved functional recovery after SCI. In this regard, RT-PCR, Western Blot and bioinformatics analysis reported that miR-21m through the inhibition of PTEN/PDCD4, suppressed neuronal death. Additionally, the therapeutic potential of miR-21 has been shown in SH-SY5Y and U251 cells transfected with MSC-EXOs transfected by miR-21 or PTEN small interfering RNA (siRNA). In addition, in vitro, the treatment increased cell viability and suppressed cell death by the miR-21/PTEN/PDCD4 signalling pathway [103]. MiR-21 is known to have the ability to phosphorylate PTEN, which is important in SCI as its silencing in SCI mice was shown to induce cortical neuron growth and increased levels of the mammalian target of rapamycin (mTOR) [104]. In addition, Wang et al. demonstrated the effects of EXOs isolated from PC12 cells or MSCs transfected with phosphatase and tensin homolog pseudogene 1 (PTENP1) short hairpin RNA (shRNA). The EXOs transfected with PTENP1 shRNA led to a reduction in apoptosis both in the SH-SY5Y and U251 cells, according to a mechanism that involved the overexpression of miR-21 and miR-19b, as well as the downregulation of PTENP1 and PTEN. Moreover, the in vitro results were also confirmed in SCI SD rats induced by a contusive model. The in vivo results confirmed the reduction in neuronal apoptosis 28 days after the transplant in the experimental groups treated with transfected EXOs. Therefore, MSC-EXOs transfected with PTENP1 shRNA, through the modulation of miR-19b and miR-21 expression, could be proposed as a new tool for post-SCI recovery [105]. The therapeutic potential of miR-21 and miR-19b derived from hMSCs-EXOs has also been highlighted by Xu et al. The in vitro results demonstrated that, after 48 h, the treatment with EXOs isolated from the supernatant of MSCs and PC12 cells differentiated, led to an increase in miR-21 and miR-19b expression, as well as a decrease in PTEN expression in SH-SY5Y and U251 cells. Therefore, treatment with PC12 cells/MSC-EXOs exerts an inhibitory effect on neuron cell apoptosis. Additionally, the authors evaluated the role of the miR-21/miR-19b pathway in an SCI rat model. The intravenous administration of PC12 cells/MSC-EXOs led to an improvement in functional recovery, as well as reduced neuronal apoptosis in SCI animals. Moreover, it has been confirmed that miR-21 and miR-19b derived from hMSCs-EXOs exerts neuroprotective effects by regulating PTEN expression [106].

Huang et al. investigated the effects of EXOs derived from miR-126-modified MSCs on SCI both in vivo and in vitro. The in vivo results demonstrated that, 28 days after injury, the intravenous administration of labelled miR-126 EXOs led to a reduction in lesion volume and improved functional recovery in SD rats induced by moderate contusion injuries. In addition, in vitro, labelled miR-126 EXOs improved the angiogenesis and migration of HUVECs through the inhibition of Sprouty-related EVH1 domain-containing protein 1 (SPRED1) and phosphoinositide-3-kinase regulatory subunit 2 (PIK3R2), i.e., two targets of miR-126 EXOs. Indeed, it was reported both in vitro and in vivo that treatment with miR-126 EXOs led to a suppression of SPRED1 and PIK3R2 expression and, conversely, an increase in VEGF. These data highlight the potential therapeutic capacity of MSC-EXOs to efficiently transfer miRNAs to the injured spinal cord, thus proving to be a novel potential therapeutic strategy for treating SCI [107].

These data demonstrate the protective role of miRNAs in regeneration and neurogenesis, highlighting their potential use as a treatment for SCI. In this regard, we investigated whether systemic injections of MSC-EXOs transfected with miR-133b, a miRNA already known for its neuroprotective effects in SCI, could reduce spinal cord damage. The study demonstrated that, five days after the transplant, the animals systemically injected with miR-133b EXOs showed an improvement in the recovery of hindlimb function compared to control groups. Moreover, both a reduction in the injury volume and the neuronal cell loss, as well as an increase in the axonal regeneration in the miR-133b EXOs group, were reported. Furthermore, four days following SCI, miR-133b EXOs increased extracellular-signal-regulated kinase (ERK) 1/2 phosphorylation by targeting Ras homolog gene family member A (RhoA) and thus avoiding neuronal cell death. In addition, miR-133b EXOs led to the activation of the signal transducer and activator of transcription 3 (STAT3) and cAMP-response element binding protein (CREB), thus promoting the neuronal cell survival and axonal regeneration; this highlighting once more the potential role of MSC-EXOs transfected with miRNAs [108]. In contrast, Jiang et al. demonstrated the effects of MSC-EXOs transfected with miR-145-5p in an SCI experimental study. The MSC-EXOs transfected with miR-145-5p led to inflammation reduction and improved the animal’s functional recovery according to a mechanism that involved the Toll-Like Receptor (TLR)4/NF-κB signalling pathway modulation. In order to confirm the MSC-EXOs effects on cell viability, apoptosis and inflammation, the authors induced an inflammation model in PC12 cells through LPS (5 μg/mL) and then incubated it with 10 μg of MSC-EXOs transfected with miR-145-5p. It is noteworthy that the Western Blot analysis demonstrated that MSC-EXOs containing miR-145-5p reduced the inflammation, as well as the TLR4/NF-κB pathway activation in the PC12 cells [109]. In line with the previous results, Lu et al. demonstrated that the transplant of BMSCs-EXOs reduced neuronal cell death and improved motor recovery through a mechanism that preserved the BSCB integrity. In this regard, it has been shown that the administration of BMSCs-EXOs suppressed the migratory potential of pericytes, thus promoting BSCB integrity via the NF-κB p65 pathway. To confirm the in vivo results and to simulate the pathological environment of SCI in vitro, pericytes isolated from SD rats were exposed to OGD/reperfusion conditions and treated with BMSCs-EVs. In addition, in vitro, treatment with BMMSCs-EVs reduced the migration of pericytes and thus preserved the integrity of the BSCB via NF-κB p65 pathway modulation [66]. It is worth noting that the NF-κB pathway has been related to A1 astrocyte activation and the inflammatory response after SCI. Therefore, with this aim, Wang et al. investigated the inhibitory effects of MSCs-EXOs on the activation of astrocytes following SCI. The intravenous administration of both MSC-EXOs and MSCs led to a decrease in A1 astrocytes activation through the inhibition of the nuclear translocation of NF-κB p65, induced by SCI in the ventral horn of the spinal cord. Moreover, it has been shown that the administration of MSC-EXOs or MSCs reduced pro-inflammatory cytokine levels, including TNFα, IL-1α and IL-1β in the ventral spinal cord after SCI, as well as a decrease in the injury area and improved functional recovery in SCI rats. Additionally, the infusion of MSC-EXOs or MSCs in SCI rats also exerts neuroprotective and anti-apoptotic effects, as shown by the increase in the Myelin Basic Protein (MBP), Synaptophysin (Syn) and Neuronal Nuclei (NeuN). In addition, the in vitro exposure to MSCs-EXOs and MSCs reduced the proportion of SCI-induced A1 astrocytes, most likely through the inhibition of NFκB p65 nuclear translocation [77].

In compliance with this study, Liu et al. demonstrated the efficacy of MSC-EXOs in the suppression of A1 neurotoxic reactive astrocytes activation post-SCI. The BMMSCs-EXOs treatment led to a reduction in A1 neurotoxic reactive astrocytes and suppressed neuronal apoptosis. Through the proangiogenic effects, 28 days after SCI, the treatment with BMMSCs-EXOs promoted axonal regeneration and functional behavioral recovery, as well as reduced glial scar formation, lesion size and inflammation. To confirm the results obtained, the authors performed several in vitro functional assays. In line with the in vivo results, the incubation of HUVEC led to the proliferation, migration and tube formation of HUVEC. The BMMSC-EXOs pre-treatment led to a reduction in the release of LPS-induced nitric oxide in the microglia after 24, 48, 72, and 96 h. Moreover, it has been reported that BMMSCs-EXOs reduced neuronal cell death in the primary neurons induced by treatment with glutamate for 24 h [110]. Taken together, these results suggest that the application of BMMSC-EXOs treatment, which represses A1 neurotoxic reactive astrocytes activation, may be a promising strategy for SCI.

The results of the aforementioned studies demonstrate the potential beneficial effects of MSCs and their MSC-exosome in models of acute SCI. As intravenously administered MSCs are known to not reach the site of injury, it has already been observed that small EVs characterized as exosomes by the trafficking of bone marrow-derived MSCs (MSC-smallEVs) reach the site of injury and target the type M2 macrophages in the injured spinal cord. In this regard, as smallEVs are smaller and more stable and storable than living cells, Nakazaki et al. hypothesized that the infusion of MSC-smallEVs could overcome the many problems associated with cell infusion. Indeed, the transplant of MSC-smallEVs led to BSCB stabilization and improved functional recovery compared to the control animal groups. Additionally, the authors observed that labelled MSCs-EVs have been associated specifically with M2 macrophages and are co-localized with exosome markers, such as CD63, in the lesion site. Moreover, it has been reported that the infusion of EVs increased the expression of M2 macrophage markers, TGF-β and TGF-β receptors, and reduced BSCB permeability. The in vitro results confirm that MSC-sEVs are only taken up by cells when polarized to an M2 phenotype with IL-4 treatment. On the contrary, the cells stimulated to an M1 phenotype did not show a detectable uptake of the EXOs. Overall, these data suggest that MSC-sEVs promote a cascade of cellular responses that improve functional recovery in SCI. Finally, MSC-smallEVs, by targeting M2 macrophages, activate the TGF-β signalling pathway, thus exerting therapeutic effects in SCI. Therefore, contrary to MSCs which could cause risks related to pulmonary embolism, the results of this study demonstrate that multiple administrations of MSC-sEV could be safe. These data highlight how the intravenous administration of MSC-sEV can represent an important non-cellular therapeutic approach for the management of this pathology [111].

In this regard, Lankford et al. evaluated the possible mechanism of action of MSC-EXOs in SCI by studying the tissue distribution and cellular targeting of 1,1-dioctadecyl-3,3,3,3-tetramethylindotricarbocyanine iodide (DiR) fluorescently labelled MSC-EXOs at 3 h and 24 h after intravenous infusion in SCI rats. The DiR labelled MSCs-EXOs were detected in the lesion site in M2 macrophages that express the marker CD206. Therefore, the data suggest that the MSCs-EXOs’ regulation of the macrophage’s action into the lesion site can contribute to the therapeutic effects of EXOs released by MSCs. MSC-EXOs targeting M2-type macrophages at the site of SCI support the idea that EXOs, released from MSCs, may mediate at least some of the therapeutic effects of the intravenous administration of MSCs [83].

To improve the therapeutic potential of EXOs as a possible clinical application on SCI, researchers are developing different strategies. At present, MSC-secreted nano-sized EXOs have demonstrated an important potential to promote functional behavioral recovery after SCI. As normoxic conditions that occur in vitro differ from the hypoxic micro-environment in vivo, Liu et al. evaluated the effects of MSCs-EXOs under hypoxic (HEXOs) or normoxic (EXOs) conditions, as well as their underlying mechanism. miR-216a-5p is most enriched in HExos and is potentially involved in the microglial polarization of HExos-mediated. The authors isolated EXOs from BMSCs cultivated in normoxic or hypoxic conditions and they transfected them with miR-216a-5p mimics or miR-216a-5p inhibitors, as well as being co-incubated for 24 h with the microglial cell line BV-2 or primary microglia, both induced with LPS to reproduce microglial neuroinflammation in vitro. Although no morphological differences have been shown in experimental groups treated with hypoxic or normoxic conditions, both a greater protein concentration and of the easy up-take of EXOs labelled by microglial cells have been highlighted in HEXOs groups. Moreover, an increase in anti-inflammatory cytokines, as well as a decrease in pro-inflammatory cytokines, both in BV2 cells and primary microglia treated with overexpressed miR-216a-5p-HEXOs (miROE-HEXOs), has been demonstrated. On the contrary, it has been reported that the knockdown of miR-216a-5p in HEXOs (miRKD-HEXOs) could reduce the beneficial effects seen with HExos. Additionally, the authors used C57BL/6 male mice induced by laminectomy and a spinal cord impactor, as well as immediately after SCI infused by tail vein injection with EXOs 200 μg precipitated in 200 μL PBS, or an equal volume of PBS (200 μL). The in vivo results demonstrated that the administration of HEXOs promoted functional behavioral recovery after SCI compared to the EXOs mice group. It has been reported that the transplant of HEXOs led to a shift in the polarization of the microglial/macrophage from the M1 to M2 phenotype. Additionally, it has been confirmed that miR-216a-5p overexpression facilitated the polarization of the microglia/macrophage from the M1 to M2 phenotype. As TLR4 has been identified as a target of exosomal miR-216a-5p, the TLR4/NF-κB/PI3K/AKT signalling pathway can be involved in the microglial polarization modulation. In conclusion, the combination of miRNAs and MSC-EXOs derived from hypoxic conditions could be a minimally invasive approach for SCI treatment [112].

Although there are numerous advantages over MSCs, both EXOs and MSC-derived nanovesicles (N-NVs) do not show the ability to target diseased organs after systemic administration. In this regard, Lee et al. developed and evaluated the effects of macrophage membrane-fused umbilical cord blood-derived MSCs (MF-MSCs). As macrophage membranes contain several binding molecules, including α4β1 integrin, as well as receptors of inflammatory cytokines, MF-NVs by neuroprotective, anti-inflammatory and angiogenic effects, they could improve the efficacy of NVs on SCI. To mimic the ischemic conditions in the spinal cord, HUVECs underwent hypoxia and MF-NVs were added. The in vitro results demonstrated a greater targeting efficiency of MF-NVs for HUVECs cultivated in hypoxic-conditioned. Moreover, the intravenous administration of MF-NVs led to an improvement in the functional recovery in SCI C57BL/6 mice induced by the compression model 1 h after injury and were injected again seven days post-injury. Moreover, the injection of NVs reduced the acute inflammation and cellular damage that occur after SCI. In addition, three days post-injury, the proliferative and remodelling phases that are involved in angiogenesis were observed. As has been reported in the previous studies, the NVs membrane coated from immunocytes, including neutrophils and macrophages, could likely remove inflammatory cytokines, such as IL1β and TNFα, so to explain the targeting mechanism of MF-NVs [113]. Thus, potentiated exosome-mimetic nanovesicles appear as potential therapeutical strategies for SCI management.

In addition, in order to improve the release and integration of EXOs, Li et al. used an innovative implantation strategy related to human MSCs-EXOs immobilized in a peptide-modified adhesive hydrogel (pGel). Otherwise from systemic treatment, MSCs-EXOs immobilized in a pGel contain exosome-encapsulated in an extracellular matrix are able to be inserted in the damaged nervous system, leading to a mitigation of the SCI microenvironment. Before performing the in vivo study, the authors evaluated the delivery capacity of MSCs-EXOs encapsulated in pGel compared to the hyaluronic acid hydrogel group. To induce the in vivo study, SD rats underwent laminectomy, and the spinal cord was transected. Following the lesion, the animals were implanted with human MSCs-EXOs (100 μg) that had been suspended in PBS (20 μL) and injected into the pGel (60 μL). The study results demonstrate the efficacy of the transplant in functional recovery and urinary protection. Finally, 28 days after the Exo-pGel transplants, the exert regenerative effects leading to lesion reduction and an increase in myelin sheaths were observed, thus explaining the improved locomotor functions [100].

**Figure 3:**
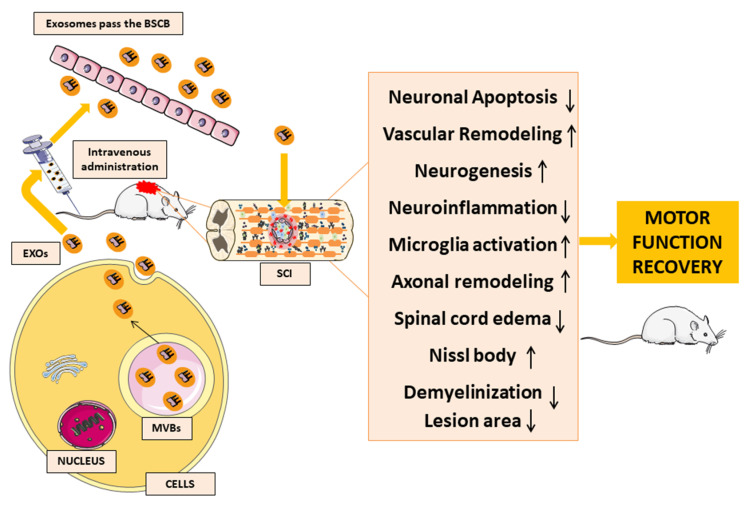
Repair of the nervous system following SCI post exosomes transplantation. Exosomes secreted by donor cells can cross to the blood-brain barrier, reducing both neuroinflammation and neuronal apoptosis, thus promoting vascular remodeling, neurogenesis, microglia activation and axonal remodeling in the nervous system. The image was created using the image bank of Servier Medical Art (Available online: http://smart.servier.com/, accessed on 1 December 2022), licensed under a Creative Commons Attribution 3.0 Unported License (Available online: https://creativecommons.org/licenses/by/3.0/, accessed on 1 December 2022).

The results of all of these studies (Table 1) show the beneficial effects of MSC-EXOs transplantation in neuron tissue regeneration and motor function recovery, as shown in Figure 3. The clinical data and experimental animal reports show that exosomes, in particular exosomal miRNAs, are closely associated with SCI. For this reason, MSC-EXOs could have potential applications in several diseases and may be a promising hope for SCI management. Indeed, MSC-EXOs can cross the BSCB, reducing both neuroinflammation and neuronal apoptosis, thus promoting vascular remodelling, neurogenesis, microglia activation and axonal regeneration in the nervous system and protecting the BSCB.

## 7. Challenges and Future Perspectives

For decades, research has focused on regenerative strategies capable of improving the recovery of the injured spinal cord after SCI. Although the transplant of MSCs seems to be a potential treatment for SCI, factors such as tumorigenesis, low survival rate and immune rejection, as well as the lack of direct differentiation of MSCs in neuronal cells, can limit their application to promote spinal cord repair. The study of MSC-EXOs could represent new challenges and opportunities for developing regenerative strategies. However, before MSC-EXOs can be applied to clinical practice, it is necessary to address several challenges related to the source and optimal culture conditions, including isolation, purification, amplification, as well as the optimal dose, frequency and route of EXOs administration. As MSC-EXOs isolated from different tissue sources contain different inclusions, they show different abilities to repair damage. Consequently, the choice of MSC-EXOs should be performed according to the different tissues of origin. Furthermore, in order to not affect the biological activity of EXOs, it is necessary to implement standardized, inexpensive, rapid isolation and purification procedures. Indeed, the different separation methods show significant differences in RNA and protein contents. Hence, the storage, transportation, and preservation of EXOs are fundamental. In order to improve the recovery of the injured spinal cord after SCI, a further challenge is represented by the sufficient MSC-EXOs number to be administered. Consequently, physiological stimuli, as well as chemical and physical pretreatment, including hypoxic preconditioning or cytokine preconditioning, could improve the paracrine effects of MSC-EXOs and regulate the production and release of different factors, as well as of EXOs. Additionally, innovative implantation strategies, such as EXOs-encapsulated in an extracellular matrix, have led to an improvement in the capacity of EXOs inserted into damaged nervous systems. Despite the PEG being the gold-standard technique for modifying the surface of nanoparticles, new techniques, such as integrated cell cloaking nanotherapeutics, are being investigated in order to improve the increment of nanoparticles in the target tissue and, thus, being used as vehicles for drug delivery, immune modulation and tissue regeneration. Although encouraging results are being obtained by methods that combine MSC-EXOs with biological materials, or those that used MSC-EXOs for targeted drug or gene delivery, future studies are needed to investigate them in depth.

## 8. Conclusions

As the therapeutic effects of MSCs are related to their paracrine activity by the release of proteins, lipids and high levels of miRNAs in their contained MSC-EXOs, modulating the signaling pathways involved in their neuro-regenerative and neuroprotective effects could be a potential tool to treat SCI. Several experimental studies have demonstrated that different miRNAs, including miR-21; miR-19; miR-126; miR-133b; miR-199a-3p/145-5p; miR-26a and miR-544, through modulation of the signaling pathways PTEN/AKT/mTOR, NF-κB p65 or ERK1/2, reduced astrocytes activation and pericytes migration suppressed both neuronal apoptosis and inflammation in SCI. Additionally, an improvement in the functional recovery induced by axonal regeneration, the release of proangiogenic factors and the polarization of macrophages toward the M2 state have been shown, thus promoting BSCB integrity and a reduction in the damage post-SCI. As, at present, there are no standardized methods to isolate and administered MSC-EXOs, it could be useful to investigate new methods to deliver the EXOs directly to the lesion site. Moreover, it is necessary to understand the additional mechanisms involved in the regenerative properties, as well as to establish the efficacy and safety of transplanting EXOs in SCI patients; hence, further studies are required.

## Figures and Tables

**Table 1:** Synthesis of the studies that evaluate the role of MSC-EXOs in several in vivo and in vitro models of SCI.

Cell Therapy	Dose	Rout of Administration	Intervention	Results	Type of Study	Ref.
hEpi ADMSCs-EXOs	1 × 10^9^ and 5 × 10^9^ particles in 0.2 mL PBS	Intravenous administration	Immediately after SCI was induced, the hEpi ADMSCs-EXOs were injected into the animals and the same amount was administered again after 3 days.	The hEpi ADMSC-EXOs injection improved SCI and reduced the inflammatory response of spinal cord injury through the regulation of various cytokines and targeting immune response and neurogenesis-related genes in the spinal cord tissue. Conversely, Increased the expression of neurotrophin factors such as BDNF and VEGF.	In vivo	[96]
BMMSCs-EXOs	200 μg/mL, ~1 × 10^6^	Intravenous administration	BMMSCs-EXOs were administered 30 min and 1 day after SCI	The BMSC-EXOs counteracted neuronal cell death, and reduced myelin loss, improving myelin disposition. Furthermore, the treatment increased pericytic/endothelial cell coverage on the vascular wall, inhibited caspase 1 and IL-1β expression, decreased blood-spinal barrier leakage, and promoted accelerated functional recovery in rats. with SCI. In addition, in vivo exposure to BMSC-EXOs it reduced pericyte pyroptosis and increased its survival rate.	In vivo	[97]
	100 μg/mL	Pericytes co-incubation and exposure to BMSCs-EXOs	Pericytes were co-incubated with or without BMSCs-EXOs for 8 h before exposure to a compound stimulus of IFN-γ + TNF-α + LPS + Lipofectamine + ATP		In vitro	
hPMSCs-EXOs	200 μg/μL	Intrathecal injection	Exosomes were directly injected by stereotactic injection into the epicenter of the SCI after damage	The hPMSCs-Exos have proangiogenic effects on endothelial cells inducing tube formation. In addition, in vivo, the hPMSCs-Exos treatment enhanced angiogenesis in the SCI rats and promoted functional recovery.	In vivo	[98]
	100 μg/mL	HUVECs SCI model exposed to hPMSCs-EXOs	HUVECs have been cultivated in OGD conditions and undergo scratch in order to induce the SCI model and subsequently exposed to hPMSCs-EXOs		In vitro	
BMMSCs-EXOs-miR-26a	20 μg/ml	Intravenous administration	BM-MSCs were transfected with the mimics of miR-26a, and the exosomes were collected. Subsequently, PC12 cells were incubated with BMMSCs-EXOs-miR-26a for 48 h.	BMMSCs-Exos-miR-26a induced neurofilament generation in vitro reducing PTEN expression and increasing the PI3K, AKT, and mTOR proteins phosphorylation. In vivo, treatment enhanced axonal regeneration, and neurogenesis, conversely, it reduced glial scarring and improved functional recovery through PTEN/AKT/mTOR signaling cascades.	In vitro	[99]
	200 μg in 200 μL PBS	PC12 cells incubated with BMMSCs-EXOs-miR-26a	Immediately following SCI, the rats received an injection of BMMSCs-EXOs-miR-26a via tail vein injection.		In vivo	
BMMSCs-EXOs containing miR-544	100 μg diluted in 0.5 mL PBS	Intravenous administration	24 h after SCI, the rat model was induced, the animals received an injection of BMMSCs-EXOs containing miR-544 via tail vein injection.	BMMSCs-EXOs containing miR-544 reduced inflammation and improved both neuronal survival and consequently promoted functional recovery after SCI	In vivo	[100]
hUCMSCs-EXOs co-transfected with miR-199a-3p/145-5p	0, 1, 3, 5, 7, 10, 13, 15, 17, 20, 25 μg/mL	PC12 cells incubated with hUCMSCs-EXOs co-transfected with miR-199a-3p/145-5p	After being pretreated with hUCMSCs-EXOs or with hUCMSCs-EXOs co-transfected with miR-199a-3p/145-5p, the PC12 cells were treated with LPS (11 μg/mL) in order to establish an injury model.	Administration of hUCMSCs-EXOs co-transfected with miR-199a-3p/145-5p to neurons of SCI rats upregulated TrkA expression at the site of injury. Consequently, the downstream pathways of NGF/TrkA and Akt were inactivated. Thus, the treatment promoted locomotor recovery in SCI rats indicating that hUC-MSC-EXOs may be a promising treatment strategy for SCI.	In vitro	[101]
	200 μg	Intravenous administration	SD rats were induced with laminectomy and subsequently underwent artery clamp to trigger acute trauma. After the SCI model was induced, the animals were injected via the tail vein with hUCMSCs-EXOs or hUCMSCs-EXOs co-transfected with miR-199a-3p/145-5p		In vivo	
MSC-EXOs isolated from obese rats transfected with miR-21	100 mg in 0.5 mL of PBS	Intravenous administration	The animals were treated with MSC-EXOs isolated from obese rats transfected with miR-21 mimic or without transfection 24 h after injury	The MSC-EXOs isolated from obese rats, due to a reduction in the levels of miR-21, as a result of insulin resistance, do not exert protective effects against skiing. On the contrary, the MSC-EXOs transfected with miR-21 Mimic showed an increase in the level of miR-21 in MSC-EXOs isolated from obese rats decreased cell apoptosis and area of injury, thus recovering their protective effects against this pathology.	In vivo	[97]
MSC-EXOs transfected with miR-21	-	Intravenous administration	Immediately following SCI, the rats received an injection of MSC-EXOs transfected with miR-21 via tail vein injection.	The MSC-EXOs transfected with miR-21 protected neuronal cells from SCI-induced apoptosis and improved the functional recovery after injury by the miR-21/PTEN/PDCD4 signaling pathway	In vivo	[98]
	-	SH-SY5Y and U251 incubated with MSC-EXOs transfected with miR-21 or PTEN siRNA	SH-SY5Y and U251 cells were firstly transfected with miR-21, or PTEN siRNA using Lipofectamine 2000 and subsequently were treated MSC-EXOs transfected with miR-21 or PTEN siRNA for 48 h		In vitro	
PC12 cells/ /MSC-EXOs co-transfected with miR-21/miR-19b	-	SH-SY5Y and U251 incubated with PC12 cells/MSC-EXOs co-transfected with miR-21/miR-19b	SH-SY5Y and U251 cells were transfected with miR-21, miR-19, or PTEN siRNA using Lipofectamine 2000 and subsequently were treated with PC12 cells/MSC-EXOs transfected with miR-21/miR-19b for 48 h.	PC12 cells/MSC-EXOs transfected with miR-21/miR-19b suppressed the apoptosis of neuron cells by downregulating the PTEN expression.	In vitro	[105]
	-	Intravenous administration	After SCI, the animals were treated with PC12 cells derived EXOs or with MSC-EXOs transfected with miR-21/miR-19 or PTEN siRNA.		In vivo	
PC12 cells/MSC-EXOs		SH-SY5Y and U251 incubated with PC12 cells/MSC-EXOs	SH-SY5Y and U251 cells were co-transfected with miR-21 and wild type/mutant PTEN mRNA or miR-19b and wild type/mutant PTEN mRNA using Lipofectamine 2000 for 48 h	In EXOs derived from MSCs and PC12 cells, the elevated expression of miR-21 and miR19b reduced PTEN expression and attenuated apoptosis in neuronal cells confirming the therapeutic effects of PC12 cells/MSC-EXOs by downregulating the expression of PTEN.	In vitro	[106]
		Intravenous administration	After SCI, the animals were treated with PC12 cells derived EXOs or with MSC-EXOs.		In vivo	
MSC-EXOs transfected with miR-126	100 μg, about 1 × 10^10^ particles diluted in 0.5 mL of PBS	Intravenous administration	Approximately 30 min after injury, the rats were treated with MSC-EXOs transfected with miR-126 or miR-con EXOs via tail vein injection	MSC-EXOs transfected with miR-126 efficiently transferred miR-126 to the site of injury, induced angiogenesis by suppressing SPRED1 and PIK3R2, stimulated neurogenesis, and protected neuronal cell to apoptosis SCI induced.	In vivo	[107]
	10 μg	HUVECs OGD model exposed to hPMSCs-EXOs	First, HUVECs were incubated with miR-con EXOs or miR-126 EXOs for 6 h and subjected to OGD. Then, the cells were incubated with MSC-EXOs for 2 hours		In vitro	
MSC-EXOs transfected with miR-133b	100 μg in 0.5 mL of PBS	Intravenous administration	The animals were treated with MSC-EXOs transfected with miR-133b, 24 h after injury	Systemic administration of miR-133b-transfected EXOs upregulated miR-133b expression at the site of injury and protected neuronal cells from apoptosis and promoted axon regeneration. Part of these effects was mediated by the activation of the ERK1/2, STAT3, and CREB pathways, targeting RhoA which is instead inhibited.	In vivo	[102]
MSC-EXOs transfected with miR-145-5p	100 μg in 0.5 mL of PBS, equivalent to 1 × 10^10^ particles	Intravenous administration	The animals were treated with MSC-EXOs transfected with miR-145-5p 30 min after injury	MSC-EXOs transfected with miR-145-5p can be useful to reduce the inflammation in SCI by the involvement of TLR4/NF-κB pathway modulation.	In vivo	[109]
	10 μg	PC12 cells incubated with MSCs-EXOs transfected with miR-145-5p	After 24 h, PC12 cells treated with LPS were incubated with MSCs-EXOs containing miR-145-5p for 48 h		In vitro	
BMMSCs-EVs	200 μg/mL	Intravenous administration	BMMSCs-Evs were injected into the tail vein 30 min post-SCI and 1-day post-injury	BMMSC-Evs reduced apoptosis in neuronal cells, promote regeneration, improved motor function, and attenuated the disruption of BSCB and pericyte coverage via NF-κB p65.	In vivo	[63]
	100 μg/mL	Pericytes co-incubation and exposure to BMSCs-Evs	Pericytes were seeded 100 μg/mL of BMMSC-Evs and after were exposed to OGD/reperfusion exposure		In vitro	
BMMSC-EXOs	200 µg/mL, derived from ∼1 × 10^6^ MSCs	Intravenous administration	30-min post-injury and 1 day after, the animals were infused by tail vain with BMMSCs or with BMMSC-EXOs	Both MSCs and MSC-EXOs administration exerted anti-inflammatory and neuroprotective properties attenuating SCI-induced A1 astrocytes activation via inhibiting nuclear translocation of NFκB p65	In vivo	[77]
	5 × 10^4^	Astrocyte co-culture with BMMSCs or BMMSC-EXOs	Astrocytes collected from SCI rats co-cultured with BMMSCs or MSC-EXOs for 48 h		In vitro	
BMMSC-EXOs	200 μg of BMSCs-EXOs precipitated in 200 μL of PBS	Intravenous administration	After SCI, the rats were treated with BMSCs-EXOs via tail vain	The BMMSC-EXOs treatment induced functional behavioral recovery, enhancing blood vessel formation, reducing glial scars, protecting the neuronal cells against apoptosis, increasing axonal regeneration, and decreasing inflammatory response by suppression of the activation of A1 neurotoxic reactive astrocytes.	In vivo	[110]
	100 μg/mL	HUVECs exposed to BMMSCs-EXOs	2 × 10^4^ HUVEC cells were seeded and after co-incubated with BMMSC-EXOs for 24 h. It has been tested the effects of BMMSCs-EXOs on nitric oxide production, HUVEC cells, 1 hour before stimulation with 5 ng/mL LPS, microglia 2 × 10^5^ cells/mL, have been pretreated with or without BMSCs-EXOs 100 μg/mL		In vitro	
BMMSC-small EVs	2.5 × 10^9^ or 8.3 × 10^8^	Intravenous administration	On day 7 post-SCI, MSCs or MSC-sEVs suspended in 1 mL DMEM were infused via the femoral vein. On 8- and 9-day post-SCI, 0.2 mL DMEM alone or 1/3 dose MSC-sEVs in 0.2 mL DMEM was infused via the saphenous vein.	Both MSC infusion and fractionated MSC-smallEVs target M2 macrophages and augment TGF-β receptors, activating the TGF-β signaling pathway, thus promoting functional recovery in SCI animals.	In vivo	[111]
BMMSC-EXOs	2.5 × 10^9^ in 0.2 mL of PBS	Intravenous administration	One week post-injury, DiR labeled MSC-EXOs were infused into the saphenous veins	Intravenous administration of MSC-EXOs rapidly traffic to the injured and associate specifically with M2 macrophages.	In vivo	[83]
BMMSC-EXOs	200 μg/mL	BV2 exposed to BMMSCs-EXOs under hypoxic or normoxic condition	LPS (1 μg/mL) was co-cultured with BV2 microglia for 24 h followed by the addition of EXOs	Hypoxia preconditioning represents a promising and effective approach to enhancing the therapeutic properties of MSC-EXOs, promoting functional behavioral recovery in the SCI model by shifting microglial polarization from M1 to M2 phenotype in vivo and in vitro.	In vitro	[112]
	200 μg of total protein of EXOs precipitated in 200 μL PBS	Intravenous administration	Mice were subjected to SCI, followed by tail vein injection of EXOs, HEXOs, miR-NCOE-HExos, miROE-HExos, miR-NCKD-HExos, and miRKD-HExos.		In vivo	
N-NVs and MF-NVs	20 μg/mL		PC12 cells and HUVECs were cultured in the media containing 500 μM of H_2_O_2_ and lipopolysaccharide in a hypoxic incubator for 24 h. To polarize macrophages into the M1 phenotype, RAW 264.7 cells were cultured in the presence of LPS for 24 h in normoxia. After, the cells were treated with N-NVs and MF-NVs (20 μg/mL) for 24 h under hypoxic and polarization culture conditions.	Compared to normal N-NVs, MF-NVs contained a larger quantity of ischemic area-targeting molecules. The MF-NVs enhanced their accumulation in the injured spinal cord. This increased reduced apoptosis and inflammation prevented the axonal loss, increased blood vessel formation, decreased fibrosis, and consequently, improved spinal cord function.	In vitro	[113]
	25 μg of N-NV in 100 μL PBS, and 25 μg of MF-NV in 100 μL PBS	Intravenous administration	N-NVs and MF-NVs intravenously injected to tail vein 1 h and 7 days post-SCI		In vivo	
Human MSC-EXOs immobilized in pGel	100 μg suspended in 20 μL PBS	Topical transplantation	After the lesion, the animals were implanted with human MSCs-EXOs and injected into the pGel (60 μL)	The human MSC-EXOs immobilized in pGel induced nerve tissue repair and functional recovery protecting bladder and kidney tissues from SCI induced-neuronal damage.	In vivo	[100]

Mesenchymal stromal/stem cells: MSCs; human epidural adipose tissue-derived MSCs: hEpi AD–MSCs; exosomes-derived MSCs: MSC-EXOs; phosphate-buffered saline: PBS; Spinal cord injury: SCI; brain-derived neurotrophic factor: BDNF; vascular endothelial growth factor: VEGF; Bone marrow MSCs: BMMSCs; interferon-γ: IFN-γ; tumor necrosis factor α: TNF-α; lipopolysaccharide: LPS; adenosine triphosphate: ATP; interleukin-1β: IL-1β; human placenta-derived MSCs: hPMSCs; Human Umbilical Vein Endothelial Cells: HUVECs; oxygen-glucose deprivation: OGD; Phosphoinositide 3-kinases: PI3Ks; phosphatase and tensin homolog: PTEN; protein kinase B: Akt; mammalian target of rapamycin: mTOR; human umbilical cord: hUC-MSCs; human umbilical cord MSCs: hUCMSCs; nerve growth factor: NGF; extracellular-signal regulated kinase: ERK; signal transducer and activator of transcription 3: STAT3; cAMP-response element binding protein: CREB; Ras homolog gene family member A: RhoA; nuclear factor kappa-light-chain-enhancer of activated B cells: NF-κB; Toll-Like Receptor: TLR; small interfering RNA: siRNA; phosphatase and tensin homolog: PTEN; Sprouty-related EVH1 domain-containing protein 1: SPRED1; and phosphoinositide-3-kinase regulatory subunit 2: PIK3R2; extracellular vesicles: EVs; EVs derived from BMMSCs: BMM-SCs-EVs; blood-spinal cord barrier: BSCB; macrophage membrane-fused exosome-mimetic nanovesicles: MF-NVs; MSC-derived nanovesicles: N-NVs; peptide-modified adhesive hydrogel: pGel; MSCs-EXOs under hypoxic: HEXOs; overexpressed miR-216a-5p-HEXOs: miROE-HEXOs; knockdown of miR-216a-5p in HEXOs: miRKD-HEXOs.

## Data Availability

Not applicable.
